# Experiences of dysmenorrhea and its treatment among allistic and autistic menstruators: a thematic analysis

**DOI:** 10.1186/s12905-023-02370-8

**Published:** 2023-05-25

**Authors:** Lauren J. Gray, Hannah Durand

**Affiliations:** 1 Division of Psychology, Faculty of Natural Sciences, University of Stirling, Stirling, Scotland; 2 Department of Psychology, School of Health and Life Sciences, Glasgow Caledonian University, Glasgow, Scotland

**Keywords:** Dysmenorrhea, Pain, Menstruation, Autism

## Abstract

**Background:**

Dysmenorrhea (i.e., period pain) is common and debilitating. Autistic people are known to experience pain differently, yet little is known about the menstrual pain experiences of autistic menstruators relative to non-autistic peers. This study aimed to explore the experience of period pain and treatment uptake for period pain among allistic and autistic populations.

**Methods:**

This study used a qualitative design and opportunity sampling approach. Thirty-seven participants (of whom 17 were autistic) were interviewed via video-conferencing software using a semi-structured topic guide. Transcriptions of interviews were analysed using Braun and Clarke’s Reflexive Thematic Analysis. Data were initially analysed together for common themes. Autistic menstruators’ data was subsequently analysed separately to elucidate the unique experiences of this group.

**Results:**

A total of six themes were constructed from the data. Initial analysis determined three themes related to experiences of period pain and treatment uptake in both allistic and autistic menstruators. Social perception of menstruation was discussed, highlighting the normalisation of pain, the taboo nature, and gendered experience of menstruation, contributing to untreated menstrual pain. Issues within menstrual healthcare were also shared, including experiences of ineffective treatment, dismissive interactions, and insufficient menstrual education. Impaired functionality was repeatedly highlighted, with menstruators detailing significant limitations to their usual functioning due to menstrual pain and ineffective treatment. Three further themes were constructed from separate analysis of data from autistic menstruators. Autistic menstruators discussed the impact of menstruation on their sensory experiences and needs, with many identifying overstimulation during menstruation. Social exclusion was discussed as a factor contributing to the experience of menstrual pain and poor treatment uptake. The final theme identified pain communication differences between autistic and allistic menstruators resulting in reports of ineffective treatment and challenges in healthcare interactions.

**Conclusions:**

Communication differences, sensory aspects, and social factors contributed to the experience of period pain and treatment uptake for autistic menstruators. The perception of menstruation within society was highlighted by allistic and autistic menstruators as influential to their pain experience and engagement with treatment. Functionality was significantly impacted by pain for this sample. The study highlights societal and healthcare factors that could be improved to ensure accessibility of support and treatment for menstrual issues.

## Introduction

Period pain is one of the most commonly experienced gynaecological issues [1, 2]. Clinically, period pain is referred to as dysmenorrhea, and can be categorised into two groups: primary dysmenorrhea and secondary dysmenorrhea. Primary dysmenorrhea is the experience of pain without an identifiable pathological cause [1], while secondary dysmenorrhea is menstrual pain accompanying a gynaecological condition such as endometriosis [3]. Period pain is characterised as cramping that originates from the uterus, or pain within the lower abdomen, back, or legs, beginning prior to, or at the onset of menstruation and lasting up to several days [4, 5]. Period pain can be detrimental to functionality and is one of the leading causes of school and work absences globally [6, 7]. Moreover, many menstruators report that period pain impacts their ability to engage in social activities and sport [8, 9]. Overall, research has found that those who experience period pain have reduced quality of life [10–13]. Despite the prevalence of period pain and its detrimental nature, it is often underdiagnosed, undertreated, and normalised as an inevitable part of menstruation [11, 14, 15].

Research literature exploring experiences of menstruation has grown considerably in recent years. However, there remains a dearth of research exploring menstrual health among individuals with disabilities. Despite being relatively understudied, there is evidence indicating the commonness of period pain in autistic populations [16, 17]. Autism is a neurodevelopmental condition, with differences in communication, interactions, and sensory experiences [18]. Literature is beginning to show higher rates of menstrual problems such as heavy periods (clinically described as menorrhagia), irregular cycles, and period pain in autistic menstruators than allistic (i.e., non-autistic) menstruators [16, 19]. However, due to insufficient research examining the relationship between autism and menstruation, it is difficult to determine why a higher prevalence of period pain may exist.

Empirical literature demonstrates several potential determinants of period pain. From a pathophysiological perspective, gynaecological disorders such as endometriosis and polycystic ovary syndrome (PCOS) may be a cause of pain for menstruators, particularly for those in later reproductive years [20]. Interestingly, there is evidence to suggest PCOS and endometriosis are common comorbidities in autistic menstruators [21, 22]. This could indicate, and explain, a greater prevalence of period pain in autistic menstruators. In the absence of clinically identifiable gynaecological disorders causing secondary pain, prostaglandins have been found to link directly to the occurrence of period pain by inducing contractions within the uterus, ultimately leading to pain hypersensitivity [14, 20, 23]. Moreover, irregular periods and a heavy flow are risk factors for painful periods, particularly in younger menstruators [4, 24]. These risk factors are often more frequently reported in disabled populations than non-disabled; however, due to limited research, it is unclear if this is the case specifically for autistic menstruators [25]. Other risk factors for period pain include lifestyle, such as exercise and diet which has been found to impact menstrual pain occurrence and severity [26–29].

Despite limited research into autistic people’s experiences of menstrual pain specifically, evidence from the broader autism and pain literature suggests differential pain tolerance may play a role. It is well established that differences in pain experience exist for autistic people compared to allistic people, with increased hypo- and hypersensitivity to pain, differences in pain expression, and heightened interoception (i.e., the perception of sensations from inside the body) reported [30–32]. Although there are no studies examining the link between autism and period pain to date, a recent study into autistic experiences of menstruation by Steward and colleagues [25] reported that menstruation can exacerbate heightened sensitivities. This suggests that differences in pain sensitivities may contribute to the increased prevalence and severity of period pain in autistic menstruators. However, further research is required to establish a clear understanding of autistic experiences of menstruation and period pain.

Various treatments exist for period pain such as medical intervention, nutritional intervention, exercise, and holistic approaches. Pharmacological therapies are among the most common treatments for period pain and include hormonal contraception and over-the-counter or prescribed non-steroidal anti-inflammatory drugs (NSAIDs), which aim to reduce prostaglandins and subsequent uterine contractions [33, 34]. The effectiveness of pharmacological therapies is debatable, with some research indicating side-effects reduce quality of life further, while the original symptoms remain unchanged or only minimally changed [35, 36]. This may be partly attributable to inappropriate choice of treatment due to lack of education on period pain and/or limited engagement with healthcare professionals about menstrual pain [33, 37]. Menstruators do not always choose the most effective pain treatments, despite similar availability and cost; a common example of this is the use of paracetamol over NSAIDs like ibuprofen [38, 39]. Nutrition and exercise interventions are less frequently used than pharmacological approaches; however, various studies have shown the effectiveness of these in reducing the intensity of pain and its interference on everyday functioning [27, 40]. Holistic and complementary treatments such as acupuncture, massage, and reiki can also be used to manage period pain [41–43]. These treatment options appear to be effective in reducing symptomology [42, 44, 45]; however, menstruators are often unaware of their suitability to treat period pain [46].

There is no literature on treatment uptake for period pain in autistic menstruators, and minimal research for allistic menstruators; therefore, there are many unknowns about menstruators’ experiences of seeking and receiving treatment. A systematic review and meta-analysis by Armour and colleagues [39] found that only 11% of adolescents with period pain sought medical treatment, which reflects an earlier study by Proctor and Farquhar [47] that found menstruators more likely to self-manage their own pain than seek treatment. Another early study by Spears and colleagues [48] found interaction with healthcare services a barrier to receiving treatment for period pain, and therefore poor treatment uptake. As evidenced, there is a lack of research investigating treatment-seeking behaviours associated with period pain in both allistic and autistic menstruators.

### Aim of study

This study aimed to explore the experiences of period pain and treatment uptake within allistic and autistic menstruators. The intention of this study was to improve understanding of experiences of menstruation and treatment-seeking behaviour, contributing to the growing literature on understanding, knowledge, and experiences of menstruation. Moreover, the study aimed to establish an understanding of experiences of menstruation and treatment uptake specifically within the autistic population specifically, where empirical literature is sparse.

## Method

### Ethical approval

Ethical approval was granted by the General University Ethics Panel at the University of Stirling (Ref: 6532, May 2022). All participants gave informed consent prior to engagement in interviews via an online form. Consent forms were stored securely and separately from the audio-recordings and transcriptions of interviews to ensure anonymity. To protect the confidentiality of participants and comply with General Data Protection Regulation (GDPR), transcripts were anonymised, and data was stored on a secure online university-affiliated system to which only the research team had access.

### Participants and recruitment

Autistic and allistic people were recruited via social media platforms (i.e., Facebook, Instagram, LinkedIn, and Twitter). Those interested in partaking contacted the researcher via email and were provided with an information sheet. The inclusion criteria stated participants should be over the age of 18 and identify as either autistic or allistic. Participants were also required to have experienced period pain at some point throughout their menstrual life, however they were not required to state whether they experienced primary or secondary dysmenorrhea. Self-diagnosed autistic people were also eligible to participate, ensuring inclusivity, given various barriers to diagnosis in females [49]. There were 37 participants in total with a mean age of 27.7 (SD: 6.7). Twenty participants were allistic with a mean age of 27.4 (SD: 6.5), while 17 were autistic with a mean age of 27.9 (SD: 6.6). See Table 1 for demographic information.

**Table 1:** Participant demographics

Participant	Neurotype	Age	Country of Residence	Participant	Neurotype	Age	Country of Residence
NAP1	Allistic	22	England	AP1	Autistic	29	UK (Undefined)
NAP2	Allistic	23	England	AP2	Autistic	26	Scotland
NAP3	Allistic	34	Scotland	AP3	Autistic	19	England
NAP4	Allistic	28	Scotland	AP4	Autistic	25	Scotland
NAP5	Allistic	28	England	AP5	Autistic	28	USA
NAP6	Allistic	25	Scotland	AP6	Autistic	31	England
NAP7	Allistic	28	Scotland	AP7	Autistic	22	England
NAP8	Allistic	27	England	AP8	Autistic	45	Scotland
NAP9	Allistic	27	England	AP9	Autistic	26	Scotland
NAP10	Allistic	49	England	AP10	Autistic	35	Croatia
NAP11	Allistic	22	UK (Undefined)	AP11	Autistic	30	Scotland
NAP12	Allistic	27	England	AP12	Autistic	23	New Zealand
NAP13	Allistic	26	UK (Undefined)	AP13	Autistic	21	England
NAP14	Allistic	29	England	AP14	Autistic	19	USA
NAP15	Allistic	19	England	AP15	Autistic	35	USA
NAP16	Allistic	27	England	AP16	Autistic	35	Northern Ireland
NAP17	Allistic	20	England	AP17	Autistic	26	England
NAP18	Allistic	21	England				
NAP19	Allistic	31	UK (Undefined)				
NAP20	Allistic	35	England				

### Study procedure

Semi-structured interviews were carried out via video-conferencing software, Zoom. This approach to qualitative interviewing allowed flexibility, where participants had the opportunity to lead the discussion, contributing information outwith topics highlighted within the interview schedule. The interview schedule was created by the researcher for the purpose of this study and included questions on experiences, knowledge, and understanding of period pain and treatment for period pain. All interviews were conducted in English. The interview schedule is presented in Table 2. The final open-ended question presented participants with an opportunity to share anything related to their experiences and knowledge of menstrual health that was not addressed during the interview. Autistic participants were also invited to share their perception of the relationship between autism and menstruation experiences. Non-directive prompts were utilised throughout interviews to gain clarity and elaboration of responses. Interviews were audio-recorded and transcribed verbatim. Interviews for autistic participants ranged from 08:08 to 27:29 min, with an average length of 16:41 min. Allistic participant interviews ranged from 08:25 min to 30:10 min, with an average of 16:57 min. The average length of the interviews was 16:50 min. Participants were debriefed following the interview and provided with the researchers' contact information should they have any questions or concerns. There was no incentive provided for participation in the study.

**Table 2:** Interview schedule

	**Wording of Question**
1	What age are you?
2	What is your country of residence?
3	How would you describe your menstrual health?
4	What is your experience of period pain?
5	What impact does/did period pain have on your day-to-day life?
6	What is your understanding of period pain?
7	Are you aware of any potential causes or triggers of period pain?
8	What treatments for period pain are/were you aware of?
9	What is your experience of seeking treatment for period pain?
10	How soon after beginning to experience pain did you seek treatment?
11	What is your experience with self-managed treatments, like buying over the counter painkillers or using heat, for example?
12	What is your knowledge or experience of alternative treatments for period pain, for example exercise, acupuncture, a TENs machine?
13	What has your experience with healthcare professionals been when seeking treatment for period pain?
14	Do you feel your period pain has been effectively treated?
15	Is there anything further you would like to contribute about periods, period pain or treatments?
16. ^a^	Is there anything you would like to contribute regarding being autistic and experiencing periods?

^a^Autistic Participants only

### Qualitative analysis

The qualitative analytic methodology utilised in this study was thematic analysis. Braun and Clarke’s [50, 51] approach to Reflexive Thematic Analysis was used, following the six phase-analysis provided:

1. Familiarising yourself with the data: All interviews were transcribed verbatim by the researcher, establishing an understanding of the data.
2. Generating initial codes: A code is an element of the data that is of interest and can be analysed in a meaningful way in relation to the research topic. A semantic approach was taken to generate codes, by extracting relevant phrases and sentences from the data, establishing recurrences throughout the data.
3. Searching for themes: Using visual representations, the relationship between codes was considered and codes were then divided based on similarity, creating themes.
4. Reviewing themes: The coded data extracts were reviewed for each theme. The validity of each theme was then reviewed in relation to the data set and the research topic, ensuring all relevant data was expressed within one of the themes, to establish an accurate representation of the data.
5. Defining and naming themes: At this point, sub-themes within themes were considered. This stage defined each theme by identifying the context and depth of each, but also as a whole in relation to the research question.
6. Producing the report: A detailed account of each theme was established within the final report.

The six-phase analysis was carried out twice: once for all data gathered, and then again for data from autistic participants only. This allowed for differences between allistic and autistic experiences, knowledge, and treatment uptake to be identified. An inductive approach to analysis was taken, whereby themes were entirely derived from the data.

### Quality and rigour

Steps were taken to ensure the study was conducted rigorously and that the data and analysis were of sufficient quality. Broad principles for assessing quality in qualitative research generally, and Reflexive Thematic Analysis specifically, were considered and adhered to during the design, implementation, and analysis phases of the research [52, 53]. The researchers met frequently during the data collection phase to reflect on the process, and during the data analysis phase to discuss and refine initial codes and themes. Reflexivity – a set of continuous, collaborative, and multifaceted approaches that enable researchers to self-consciously critique, appraise, and evaluate how their subjectivity and context influence their research [54] – was practiced throughout the research process. Reflexive writing was utilised to document the researchers’ perspectives and decisions, creating a record that could be referred to during subsequent phases of the research. Collaborative reflexivity was also practised, whereby the research team challenged one another on assumptions and decisions from their own perspectives.

## Results

A total of six themes were identified relating to the experience of menstruation and treatment uptake. Initially, analysis of all data determined three themes (1–3). A further three themes (4–6) were identified while analysing data from autistic menstruators separately, in relation to their experiences of period pain and uptake of treatment. Table 3 illustrates the themes and subthemes derived from the data. Each theme is also described below. To ensure anonymity, quotes labelled with “AP” and a numeral identify quotes from autistic menstruators, while quotes labelled with “NAP” and a numeral identify quotes from allistic menstruators.

**Table 3:** Themes and subthemes

Theme	Subthemes
1.Social Perception of Menstruation	1.1Normalised Pain 1.2Experience of being female 1.3Menstruation is Taboo
2.Menstrual Healthcare and Education	2.1Ineffective Treatment 2.2Dismissive Interactions 2.3Menstrual Education
3.Impaired Functionality	
4.Sensory Impact of Menstruation	
5.Social Aspect of Menstruation	
6.Pain Communication	

### Theme 1: Social perception of menstruation

Menstruators highlighted that the societal perception of menstruation impacted their experience of period pain and treatment uptake.

#### Subtheme 1.1: Normalised pain

Menstruators discussed the normalisation of period pain in relation to their experiences. Some menstruators highlighted the normalisation of pain within medicine, while others mentioned the social expectation to cope with period pain. All participants discussions reflected a shared experience of normalisation of period pain within society and healthcare.

“It’s just normalised in medicine, like, ‘well yeah, you’re a woman, what do you expect?’” (NAP1).

“I think my experience, as I said, it was just something that’s there and you have as a person who menstruates, and you just need to live with it.” (AP9).

“I guess when I was younger there was a lot of shame attached to the pain because I felt like I should just be able to get on with it even though it was really sore.” (AP11).

#### Subtheme 1.2: Experience of being female

A glimpse of the experience of being female, suffering with period pain, and seeking treatment was given by participants. Many participants identified an expectation to display socially acceptable behaviour around their pain experience because of their biological sex. This links closely to the normalisation of period pain and indicates a wide societal issue that is contributing to the suffering of menstruators.

“I think speaking really broadly, in society it’s seen as something that just occurs for women, and they need to deal with it… and of course, we know that women’s pain is dismissed regularly in healthcare settings and society generally.” (NAP9).

“I think culturally, I think especially people without uteruses who haven’t experienced it, they tend to not really believe that pain. Especially, if that person looks like a woman. Women are often disbelieved by people or seen as over-exaggerating, so I think it’s probably not taken as seriously.” (AP17).

#### Subtheme 1.3: Menstruation is taboo

Both allistic and autistic menstruators discussed the taboo nature of menstruation and period pain. As in previous sub-themes, participants reiterated the engrained societal beliefs around menstruation, highlighting not only that menstrual pain has been normalised and is an innate female experience, but also that is an experience that should not, and has not been talked about for generations.

“Periods and menopause are such taboo subjects…” (AP8).

“I think culturally, it’s not talked about I don’t think, it’s very much a taboo topic, do you know what I mean? […] Nobody talks about it, it’s very hush hush.” (NAP7).

### Theme 2: Menstrual healthcare and education

Menstruators who had sought medical support for their period pain described unsatisfactory healthcare experiences.

#### Subtheme 2.1: Ineffective treatment

Many allistic and autistic menstruators expressed that they felt their period pain was not treated appropriately when seeking medical treatment. Many participants reported the treatment of period pain during teenage years to be unsuitable, in addition to reports of unsatisfactory experiences of treatment-seeking. Participants also stated that they felt medical treatment overlooked the cause of the period pain.

“It wasn’t appropriately treated by the pill. It wasn’t a very good assessment at the time. Perhaps advice and being told what to expect would have been more beneficial.” (NAP3).

“At first, other pain management avenues weren’t explored… the doctor saw me for about ten minutes, and he was like, ‘contraception is going to solve all your problems, here you go,’ and I just took his word for it.” (NAP8).

“I have tried lots of different things, but they have never cared to try and look into the cause of it.” (AP12).

“My approach was to seek an exploration for my period pain because I felt that having had the contraceptive pill, it was a bit of an aimless medication since it was to alleviate the pain, but it didn't make the cause go away or make the problem, the root problem go away.” (AP7).

#### Subtheme 2.2: Dismissive interactions

Almost all participants referred to their experiences of seeking medical treatment for period pain as dismissive. Many also explained that their attempts at seeking treatment for period pain had been impacted by previous dismissive interactions. The final excerpt presented in this subtheme poignantly highlights the negative experience of one menstruator, which reflects the experience of many participants.

“You are dismissed all the time and not taken seriously and made to feel like a silly little girl who doesn’t know exactly what their body is going through.” (AP11).

“They just have basically never taken me seriously and I’ve been trying to get this sorted for seven years.” (AP12).

“I don’t really bother going to the doctors about it anymore because they kind of brush off women’s health quite a lot.” (NAP18).

“From that first consultation with the GP [general practitioner] to actually where I am now, I just feel as though the whole time I’ve never been taken seriously at all, and I feel as though there’s been a total lack of understanding and empathy from the doctors because they just… it’s all just about trying treatments; this is the guidelines… we’ll do that… but they don’t actually think of the person, they don’t actually think of the patient.” (NAP7).

#### Subtheme 2.3: Menstrual education

As well as the taboo, participants discussed their lack of knowledge surrounding menstruation, acknowledging the need for improved education. Education on menstruation was raised by both allistic and autistic menstruators. Many participants drew a link between the taboo nature and lack of education surrounding menstrual health.

“I think a lot of issues could be addressed through education too, for example I hardly know the basics of my own cycle and what I know is from basic biology at school… Especially in school, there was nothing and I needed that. It was taboo at school, and it was too awkward to talk about it so I learned nothing, and I could have benefited from learning.” (NAP1).

“I would say I still need to learn a lot but at the same time, I don't feel like there is enough [information].” (AP7).

### Theme 3: Impaired functionality

Both allistic and autistic menstruators explained their functionality was impaired by period pain. Participants overwhelmingly described their experience of period pain as “*debilitating.*”

“Every month I get debilitating pain and I have done since the age of 11.” (AP4).

“I have a lot of period pain starting really quite young, debilitating pain at times.” (AP8).

“Just debilitating pain every month, really bad pain, really heavy… it went downhill in my teens to be honest.” (NAP7).

It was commonly reported by menstruators that their pain prevented them from functioning as they would at other times in their menstrual cycle, highlighting the severity and impact of their pain.

“I can’t do any of my activities at all on my period… I just stay in my bed.” (AP14).

“The first couple of days I can’t really do anything, so it does impact greatly.” (NAP17).

“On days when I am in pain, I might need to spend the afternoon in bed. So, it definitely does restrict my activities, absolutely.” (AP16).

### Theme 4: Sensory impacts of menstruation

Autistic menstruators highlighted the sensory impacts when menstruating and experiencing menstrual pain. Some autistic menstruators discussed the impact of heightened interoception on their period pain experiences. Sensory overload was also described by numerous autistic participants in relation to their experience of menstruation and period pain.

“I feel like when I experience things in my body, I experience it quite intensely… things like period pain and all of that, I think can have quite an impact on my mood and my anxiety because something is happening within my body and its sort of out of sorts, I struggle to remove my focus from it… it becomes a focus for me until it is done.” (AP16).

“I feel like I feel everything that is happening, and I feel like it’s just a lot of overwhelm. It might not be the worst pain ever, but I can’t get my mind off of it. Just feeling every bit of bloating, feeling everything, my clothes don’t fit right, and, you know, the skin changes, everything…” (AP15).

“I think having periods as an autistic person is really overstimulating… it takes so much energy… period pain, and the bloating, and the back pain, and the breast pain, and the joint aches, it’s just super overstimulating.” (AP5).

“Any big change in my body does overwhelm me, I think it’s something that not everyone gets and like the sensory aspect of it [period pain] is obviously a lot worse… than it is for other people.” (AP6).

### Theme 5: Social aspect of menstruation

Autistic menstruators discussed the social factors which affected their experience of period pain and treatment uptake. Some menstruators highlighted the role of social exclusion in their understanding and experience of period pain. The cruciality of social inclusion for generating comparisons and developing an understanding of typical menstruation experiences was highlighted, with autistic menstruators describing challenges in creating a frame of reference. The distress caused by a lack of context to menstruation was also discussed, relating to the taboo nature of menstruation previously identified.

“The social isolation that comes with not fitting in with anyone means that you have no frame of reference for whether your experiences are normal.” (AP1).

“I just remember desperately trying to find someone else, asking, ‘how painful would you say this is out of 10?’” (AP4).

“I feel like there is a link between the autism and the way that I experience my period, because it was really distressing for me when I started developing… it was so distressing for me that change. I had no context to it.” (AP11).

### Theme 6: Pain communication

Autistic menstruators highlighted that pain communication played a role in their experience of seeking treatment for period pain. The challenges of communicating pain were discussed, with many describing pain communication as a barrier while seeking medical treatment for period pain, particularly due to differences in expression of pain and perceived pain tolerance.

“Describing your pain, how do you know how bad your pain is, how do you know how it compares to other people’s? So that’s very difficult…” (AP1).

“I’m not very good at describing how pain is or how any experience is when I am not right in the middle of it. Like they’ll say to rate it, but I’ll say I don’t know because I am not in the middle of it or in that level of pain right now. I find it hard. Also, if I do go and I am in pain, I don’t display pain like how they think I should display pain. I deal with it very internally, so they’ll say I don’t seem like I am in pain, ‘you seem fine,’ but I am not fine.” (AP6).

“I also don’t know that I communicate effectively to medical providers… I get really nervous before I go into any of those types of appointments and although I am educated and well-spoken, I usually end up stuttering and I am not as well-spoken as I know I can be. I think sometimes by the time I get there, trying to talk to them about something, something that has probably been stewing for quite a bit, I think I can sound a little bit hysterical by the time I get to them, which unfortunately just doesn’t make a good case.” (AP8).

## Discussion

### Summary of findings

The current study aimed to explore the experiences of period pain and treatment uptake among allistic and autistic menstruators. Both allistic and autistic participants highlighted societal perceptions of menstruation as taboo and period pain as women’s burden to bear as detrimental to their experiences of managing pain and engaging with treatment. Participants described disappointing interactions with healthcare professionals during attempts to seek support for managing menstrual pain, and further cited a lack of menstrual health education as a barrier to developing effective pain self-management strategies. Functional impairment due to pain during menstruation was commonly reported by participants, which may have negative impacts on educational and professional attainment as well as quality of life. Autistic participants further described unique sensory aspects of their menstruation, notably increased interoception. Social exclusion made it difficult for some autistic menstruators to form frames of reference for what constitutes a “normal” period. Finally, challenges with pain communication and differences in pain expression were presented as barriers to accessing treatment. Cumulatively these findings extend the growing literature on menstrual pain, and, crucially, lay the groundwork for future research to better understand menstruation experiences of and develop pain management supports for autistic menstruators.

### General experiences of menstruation

The findings of this study highlight the role societal perceptions of menstruation play in the experience of menstruation, period pain and treatment uptake. Literature highlights that period pain is often underdiagnosed, as well as normalised by society as a natural part of menstruation [14, 39, 55, 56]. Participants reported two key circumstances that impact their experience of period pain and engagement in treatment options. Firstly, a gendered experience was commonly described, where period pain was trivialised due to being a “female problem.” Both autistic and allistic menstruators described their gendered experience of period pain as a barrier to accessing suitable treatment, due to established beliefs that they had to endure the pain, and that they would be dismissed if they sought medical treatment. The normalisation of period pain was also discussed by many menstruators as an expectation of menstruating, and subsequently as a barrier to treatment uptake. These findings reflect existing literature that indicates menstruators primarily seek self-directed treatments like over-the-counter medication, rather than medical treatment, due to the expectation that they should be able to cope with the pain they experience [39, 57, 58]. Menstrual healthcare was frequently discussed, whereby allistic and autistic participants were disappointed with the care they received when seeking treatment for period pain. Many participants recalled interactions where they felt dismissed and misunderstood by medical staff. This is consistent with existing literature on healthcare interactions for reproductive health concerns, especially for females [59–61]. The use of ineffective or undesired treatments was also highlighted by many participants, with hormonal contraception frequently cited as the only option given by medical professionals. Despite evidence of improvements to period pain symptoms, use of contraception may be unsuitable for some menstruators due to common side effects [62, 63] or a preference not to eliminate the monthly period [55]. Moreover, participants felt a sufficiently thorough assessment of their period pain was not carried out to identify a cause of symptoms, or to determine a more appropriate treatment than menstrual suppression through hormonal contraception. This may also highlight a lack of appropriate clinical tools to assess period pain. Together these findings indicate increased education for healthcare professionals on the causes, treatment approaches, and impacts of period pain is warranted. Furthermore, exploration of the experiences of healthcare professionals in assessing and treating individuals with severe menstrual pain may elucidate the resources they need to optimise menstrual healthcare.

It is commonly acknowledged that menstruation is a taboo subject, which contributes to gender inequality, poor understanding, and undignified and uncomfortable menstruation experiences [64–67]. This study contributes further evidence of the taboo nature of menstruation and the lack of education surrounding menstrual health. Many autistic and allistic participants reported that they felt unable to talk about periods and period pain, with some participants highlighting cultural differences around menstruation. Furthermore, participants frequently mentioned the lack of education they received on menstruation and menstrual health. This links directly to Theme 2 (Menstrual Healthcare and Education) where poor education contributes to a lack of awareness about what is normal to experience as a menstruator [8, 68, 69]. Moreover, the narrative of menstruators in this study indicates that poor menstrual health education also acts as a barrier to treatment uptake, as many menstruators are unaware of treatment options besides self-managed over-the-counter medication. This highlights the impact of menstruation as a taboo subject, and the need for further research into the evaluation of menstrual health education and awareness within societies and cultures.

Empirical literature shows that period pain can have detrimental impacts on many aspects of life, particularly functional ability. This study produced similar findings, whereby both allistic and autistic menstruators described their pain as *“debilitating.”* The severity of period pain is often downplayed within society; however, this is not the first study to illustrate menstrual pain interference with daily life [7, 70, 71]. Many participants reported that they felt unable to engage in usual activities and had to remain in bed due to their symptoms. Differences in pain perception may explain some of the variability on pain severity and disability ratings in the literature. Though it is well established that autistic people may have a hypersensitivity to pain, and often rate the intensity of pain higher than allistic people [32, 72], our study appeared to show similar descriptions of period pain for both allistic and autistic menstruators. Further research is required to determine the comparability of experiences of period pain in allistic and autistic menstruators in order to develop the most appropriate pain management supports for diverse groups.

### Autistic experiences of menstruation

For autistic people, research is beginning to identify a link between hormonal fluctuations and difficulties such as sensory challenges and emotional dysregulation [25]. This study illustrates further the impact of hormonal fluctuations, particularly during menstruation, for autistic people. Many autistic participants expressed heightened sensory sensitivities during menstruation and when experiencing period pain, causing overwhelm and overstimulation. This directly links to the theme of impaired functionality, where the experience of menstruation for many autistic menstruators reportedly consumed their thoughts and feelings during that time, reducing productivity and quality of life. Autistic menstruators also discussed the relationship between heightened interoception and menstruation. Heightened interoception is common in autistic people [30–32], and the findings of this study evidence a reported relationship between interoception and period pain experiences in autistic menstruators. This link could also be used to suggest a higher prevalence of period pain within autistic populations, which currently due to a lack of research, is undetermined [25].

Social exclusion can be a common experience for many autistic people due to communication differences and social challenges [73]. The findings of this study highlight a relationship between menstruation and social communication as experienced as an autistic person. Autistic menstruators described lacking a framework of reference regarding menstruation and period pain, whereby they were unable to gauge whether their experience was normal. Autistic menstruators reported that, due to social exclusion, they felt unable to make comparisons to peers’ experiences of period pain, leaving them confused about the menstruation experience. This novel finding may also contribute to our understanding of treatment-seeking behaviours, whereby autistic menstruators’ relative naivety regarding typical menstruation and pain experiences may have resulted in less treatment uptake. Further research investigating the implications of social exclusion on autistic menstruators is required to determine the extent of the relationship.

There is some literature that suggests autistic people may underuse treatments for period pain [16]; however, research exploring barriers and facilitators to treatment uptake is lacking. This study provides some insight into treatment-seeking behaviours in autistic menstruators. Many autistic participants discussed challenges in communicating their pain, identifying communication as a barrier to accessing adequate treatment. Moreover, autistic participants also highlighted that their expression of pain may present differently from typical pain responses. Existing literature examining pain experiences in autistic children and adults has shown internalisation of symptoms as a common response to pain, thereby presenting an atypical response to both acute and chronic pain [72, 74, 75]. This is the first study to identify that period pain expression may also differ in autistic people versus allistic people. This difference in pain expression was reported as an obstacle to treatment uptake, contributing to dismissal from healthcare professionals. These findings highlight the need for neurodiversity education within healthcare to ensure suitable medical treatment is accessible to those with diverse presentations.

### Limitations and future research

This study provides an important insight into the experience of period pain and treatment-seeking within both allistic and autistic populations. There are, however, some methodological limitations that should be considered. There was some variation in the length of the interviews, some of which were notably short. These shorter interviews may have produced less rich data compared to considerably longer interviews [76]. Despite the relatively brief interview duration, the dataset produced was suitably rich in terms of breadth and depth to address the research aim; the extent of sample specificity was suitably dense; and the quality of the dialogue was strong and focused. Together this suggests the sample holds sufficient *information power*, as conceptualised by Malterud and colleagues [77], to make a meaningful contribution to this literature.

Sample characteristics also warrant consideration. Participant demographics between the autistic and allistic group were not matched, therefore comparison between groups is limited. The findings may not be as representative of allistic menstruators, as all participants were from the UK, while autistic participants resided in several countries across the globe. Moreover, comparability of access to healthcare within different countries as well as treatment guidelines for period pain within healthcare systems is an important consideration. It is important to consider the representation of the current sample, of which a large proportion could be identified as Western Educated Industrialised Rich Democratic (WEIRD) people. The findings of the study may therefore lack insight into cultural differences in attitudes and experiences of menstruation. Additionally, the current sample also mainly consisted of well-educated autistic individuals; therefore, the findings are not representative of all menstruators on the autism spectrum. This is a common bias within research due to recruitment and methodological barriers to participation for many autistic people across the spectrum [78]. Future research with greater resources than were available within the context of this study should endeavour to maximise inclusivity so as broad a range of voices as possible can be represented.

The inclusion of self-diagnosed autistic menstruators acknowledges existing literature that highlights the barriers to diagnosis for females [49]. However, participants were not required to state whether they were self-diagnosed or formally diagnosed; therefore, it is not known if a difference in experience of period pain and treatment-seeking between diagnosed and self-diagnosed autistics exists. This could be a consideration for future research.

### Reflexivity

It is important to critically reflect on the impact of the researcher on the study and interpretation of the data, especially in health-related studies [79]. The interpretation of the data will be subjective to the sole researcher and therefore, other researchers may understand the data differently. As the lead researcher is autistic, undoubtedly the study has been shaped by this. It is likely that the experiences of autistic menstruators reflected in this study may be better understood than studies led by non-autistic researchers [80]. Additionally, it is likely that other personal characteristics may impact the research, such as the researcher also being a menstruator and identifying as female. This may have generated greater rapport between the researcher and participants, potentially improving the quality of the data gathered [81].

## Conclusion

Limitations notwithstanding, the current study makes and important contribution to the field of menstrual health research. This study is one of the first to explore experiences of period pain in autistic menstruators, and ultimately, offers insight into the experience of period pain and treatment uptake within allistic and autistic populations. Communication differences, sensory aspects, and social factors were all themes highlighted by autistic menstruators that play a role within the experience of period pain and treatment uptake. Future research should further examine treatment uptake for period pain across populations. Moreover, research on healthcare experiences within autistic populations should be expanded, to contribute to the development of interventions designed to improve access to healthcare for autistic people. Societal acknowledgement and improved education on menstrual health should be strived for to improve the experiences of period pain and treatment-seeking for allistic and autistic people.

## Data Availability

Data supporting the present findings are not publicly accessible due to ethical responsibilities for data protection. Pseudonymised data, however, may be available on reasonable request to the corresponding author.

## Abbreviations

AP: Autistic participant
GDPR: General Data Protection Regulation
GP: General Practitioner
NAP: Non-autistic participant
NSAIDs: Non-steroidal anti-inflammatory drugs
PCOS: Polycystic ovary syndrome
WEIRD: Western Educated Industrialised Rich Democratic
